# Years of life lost (YLL) from cancer is an important measure of population burden – and should be considered when allocating research funds

**DOI:** 10.1038/sj.bjc.6602321

**Published:** 2005-01-11

**Authors:** N G Burnet, S J Jefferies, R J Benson, D P Hunt, F P Treasure

**Affiliations:** 1 Department of Oncology, University of Cambridge, Addenbrooke's Hospital, Hills Road, Cambridge CB2 2QQ, UK; 2 Oncology Centre, Addenbrooke's Hospital, Hills Road, Cambridge, CB2 2QQ, UK; 3 East Anglian Cancer Intelligence Unit, Box 111, The Clinical School, Addenbrooke's Hospital, Hills Road, Cambridge CB2 2SP, UK

**Keywords:** years of life lost, mortality, research spending

## Abstract

Recently, cancer mortality has been compared to research spending by the National Cancer Research Institute (NCRI), whose research budget is approximately £250 million. The analysis shows a mis-match between mortality and research spending. As well as crude mortality rates, other measures of cancer burden should be considered because they contribute additional information. ‘Years of life lost’ (YLL) summed over each individual dying after a diagnosis of cancer represents a population-based mortality indicator of the impact of that disease on society. Years of life lost divided by the number of deaths for each cancer site produces an additional statistic, the average years of life lost (AYLL), which is a measure of the burden of cancer to the individual patient. For 17 cancer sites where data are available, four tumour sites have a rather large difference in mortality, comparing YLL to crude mortality. Years of life lost shows the population burden from cancers of the ovary, cervix, and CNS to be rather larger than suggested by crude mortality, despite screening programmes for cervix cancer. Using YLL, the underprovision of funding for lung cancer research is similar to that reported using percentage mortality. Breast cancer and leukaemia receive a relatively higher research spend than the population burden of these cancers, and the spending on leukaemia is quite extreme. Prostate cancer has a low per cent YLL but attracts a moderate amount of research spending. The use of AYLL as an indicator of individual cancer burden considerably changes the ranking of the mortality from different tumours. The mean AYLL is 12.5 years. Prostate cancer has the lowest AYLL, only 6.1 years; brain tumour patients have the highest, at just over 20 years. Comparing AYLL to research spending suggests four ‘Cinderella’ cancer sites with high individual cancer burden but low research spending: CNS tumours, cervix and kidney cancers, and melanoma. Breast cancer and leukaemia have roughly average AYLL but a considerable excess of research spending. YLL emphasises the discrepancy between research spending and mortality, and may be helpful for decisions concerning research support. Avearage years of life lost measures the burden to individual patients and may be helpful where individuals’ needs are relevant, such as palliative care. As well as crude mortality, more subtle and comprehensive calculations of mortality statistics would be useful in debates on research funding and public health issues.

Measures of mortality from all types of diseases are clearly important for public health considerations. With regards to cancer mortality, data are important to monitor the effects of screening programmes, efforts at earlier diagnosis and treatment, and the effects of environmental and other causative factors such as smoking. Recently, mortality from cancer has also been compared to research spending by the National Cancer Research Institute (NCRI) as a means to gauge the appropriate relative level of expenditure for different tumour sites, and to inform debate about the distribution of funds (BMJ News, 2002; NCRI, 2002).

The latest figures on cancer mortality from Cancer Research UK (Cancer Research UK, 2004) suggest that in the last decade (1993 to 2002) there has been an overall reduction in cancer mortality rates in both men and women. This has been achieved by a reduction in mortality from the majority of cancers, including those which are most common. There are, however, some noticeable exceptions to this, principally affecting men with melanoma, kidney cancer, oesophageal cancer and tumours of the brain and CNS. None of these tumour sites has attracted more than 3% of NCRI research spending, despite the increase in mortality associated with these tumours.

There have been valuable efforts by the NCRI to review funding in relation to mortality, leading to the publication of a strategic analysis (NCRI, 2002). This strategic analysis clearly shows a mis-match between mortality and research spending, particularly for lung cancer which is under-resourced, and for leukaemia which appears to be over-resourced. The overall research budget is approximately £250 million (BMJ News, 2002; NCRI, 2002) so the analysis has important implications, both for the NCRI overall and for researchers on individual tumour types. Within the context that mortality rates might influence cancer spending, it is important to consider the details of the measure of the mortality used. As well as crude mortality rates, other measures of cancer burden should be considered because they contribute additional, potentially valuable information.

## METHODS

Simple percentage mortality expresses the number of deaths from an individual tumour type divided by the total number of deaths from cancer as a percentage. However, another important mortality statistic that complements crude mortality indicators is ‘years of life lost’ (YLL). This is calculated for each individual dying after a diagnosis of cancer by subtracting the actual survival since diagnosis from the individual's life expectancy at the time of diagnosis, based on appropriate life tables. The YLL for a specific cancer site in a population is obtained by summing all the individual years of life lost through deaths from the cancer of interest, in that population in a specific time period. This can then be expressed as a percentage, that is, the years of life lost from a specific cancer as a percentage of the total number of years of life lost from all cancers.

Therefore, YLL is a population-based mortality indicator, which gives more weight to those diseases that kill early or are incurable. Thus, weighting is given to cancers that affect patients at a young age and who die as a result. It also counterbalances the weighting given to common cancers in older patients by simple percentage mortality. This combination of factors allows YLL to give an indication of the impact of the disease on society, that is, it represents the population burden for individual cancers.

### Years of life lost

Data from the East Anglian Cancer Registry for the 5-year period 1990 to 1994 were used to calculate YLL. No cutoff for age was used for the calculations. Although a cutoff at age 70 years has been suggested (Romeder and McWhinnie, 1977), censoring patients over 70 years is clearly unhelpful in the context of cancer mortality. Years of life lost was evaluated for the population with cancer, irrespective of whether or not they died from the cancer. The statistics used for the calculation of life expectancy were obtained from the 1990 East Anglia OPCS Life tables.

Data for 17 tumour types are available, which account for 84% of the total YLL from cancer. The figure for colo-rectal cancer also includes anal tumours. Specific YLL data for liver cancer, and head and neck cancers are not available but they represent only 2% each of simple mortality (NCRI, 2002). Unspecified ‘other’ tumour types account for the remainder of cancer deaths, but these cannot be specifically addressed in comparison to per cent YLL because of the lack of detail.

### Average years of life lost

Figures for YLL can be used to produce an additional mortality statistic, the average years of life lost (AYLL). Average years of life lost is simply an average derived by dividing YLL by the actual number of deaths for each cancer site, over the defined time period. This parameter is interesting because it provides a measure of the burden of cancer to the individual patient, rather than the population as a whole. Effectively it shows, on average, how much a patient's life is likely to be shortened by their cancer.

## RESULTS

### Years of life lost

**Figure 1:**
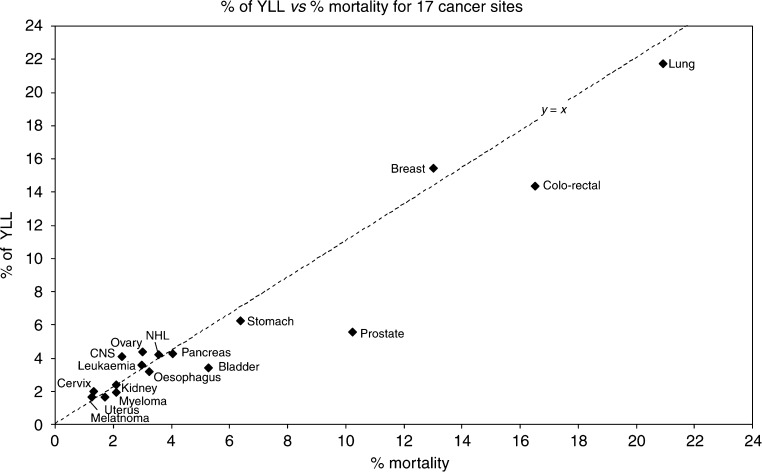
Years of life lost (YLL) *vs* crude mortality. This shows the difference between the two measures of deaths from cancer: YLL represents the population burden from a specific cancer rather than the simple percentage of deaths from that tumour. The line of equality (*y*=*x*) is shown, so that cancers whose population burden exceed their simple mortality are shown above and to the left of the line. See also Table 1.

The YLL for an individual cancer site can be expressed as a percentage of the total years of life lost from all cancers, so it can be directly compared with percentage mortality. Figure 1 shows a simple plot of percentage of years of life lost *vs* percentage mortality, for 17 cancer sites where YLL data are available. The majority of points lie close to the line of equality. This graph shows absolute differences in percentage for the two mortality indicators. Since most of the tumour sites lie relatively close to the origin, the absolute differences between them are quite small. Therefore, a helpful way to express the difference between per cent YLL and per cent mortality is to show the difference as a ratio.

Table 1 shows the percentage mortality and percentage of YLL, as well as the ratio per cent YLL divided by per cent mortality, arranged in order according to this ratio, for the 17 specified sites. The figures are normalised to sum to 100% for these sites. Several tumour types have very similar death rates with either mortality indicator, and a number of others have relatively little difference between the two measures. However, four tumour sites have a rather large difference, with ratios <0.60 or >1.4, which is not apparent from Figure 1. The population burden from prostate cancer is rather less than that is suggested by per cent mortality. On the other hand, the population burden of cancers of the ovary, cervix and CNS is rather higher, despite the presence of a screening programme for cervix cancer. Lung cancers are represented similarly by percentage YLL and percentage mortality, so the ratio is close to unity.

**Figure 2:**
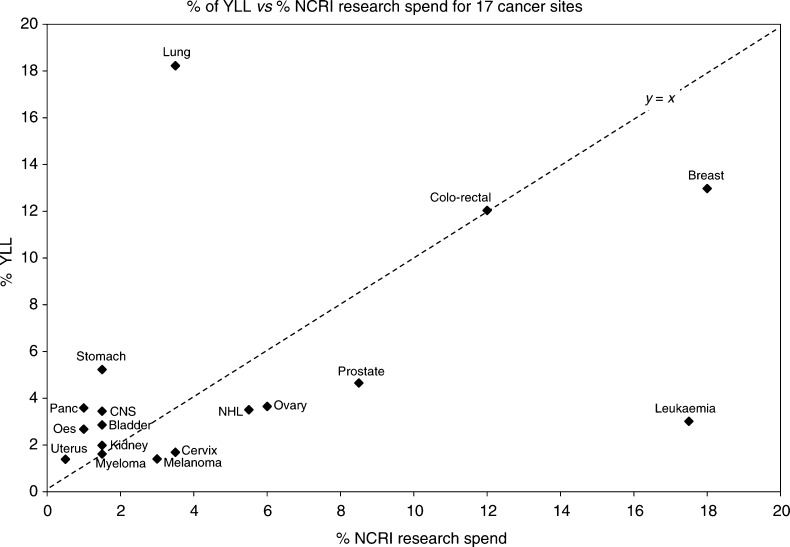
Years of life lost (YLL) *vs* National Cancer Research Institute (NCRI) research spending. The line of equality (*y*=*x*) is shown. The considerable difference between population burden and research spending for some cancers is clear. The positions of breast cancer and leukaemia to the right of the figure indicate a relatively higher research spend than the population burden of these cancers, and the relative spending on leukaemia is quite extreme. Conversely, lung cancer attracts rather less research spending than its cancer burden. Assessing this ratio for the tumour sites near the origin of the graph is better done numerically – see Table 2 for details.

In Figure 2, the percentage of YLL has been plotted against the percentage of NCRI research spend, for the 17 cancer sites. In order to make comparison more informative, the figures for YLL are now expressed as a percentage of the total YLL attributable to cancer. The 17 specific sites account for 84% of YLL from cancer. Tumour sites above and to the left of the line of equality have greater YLL than research spend, while those below and to the right have higher research spend relative to YLL. The positions of breast cancer and leukaemia to the right of the figure indicate a relatively higher research spend than the population burden of these cancers, and the relative spending on leukaemia is quite extreme. Prostate cancer has a lower per cent YLL but apparently attracts a moderate amount of research spending. Conversely, lung cancer attracts rather less research spending than its cancer burden. Using YLL as the relevant mortality indicator, the considerable underprovision of lung cancer research funding remains.

The diagram shows the outlier tumour sites clearly, but as with Figure 1, a large number of tumour sites fall near the origin and are not clearly distinguished. The data are therefore presented in Table 2, where the ratio of the two parameters indicates the similarity or discrepancy between them.

### Average years of life lost

The use of AYLL to indicate the impact of a tumour type on individual patients considerably changes the ranking of the mortality from different tumours (Table 3). The mean AYLL amounts to 12.5 years, indicating the numerical average of the life shortening from cancer. Prostate cancer, for example, has less impact on this because it affects relatively older men and can run an indolent course. In fact, it has the lowest AYLL of any of the tumours shown, only 6.1 years. Brain tumour patients, however, suffer more than three times as much loss of life, with an AYLL figure of just over 20 years.

**Figure 3:**
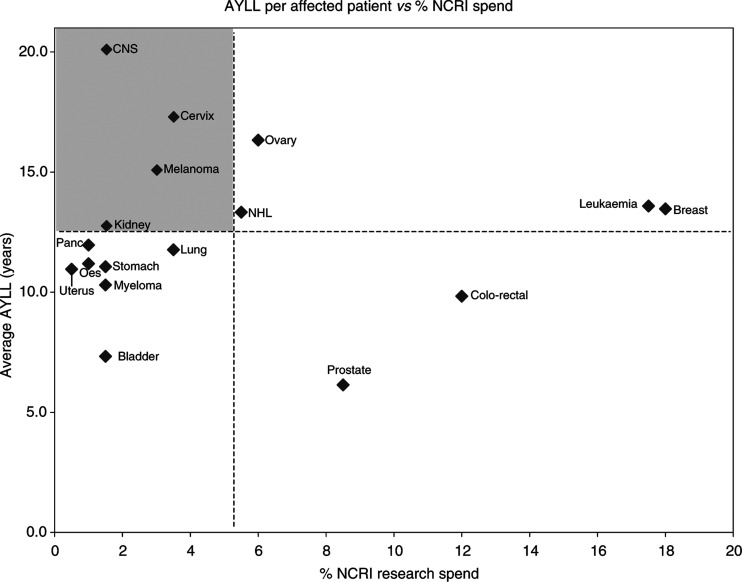
Average years of life lost (AYLL) plotted against percentage of National Cancer Research Institute (NCRI) research spending. The dashed lines show the mean percentage of NCRI spending (%) and the mean AYLL (12.5 years), for the tumour sites shown. The ‘Cinderella’ cancer sites, with the highest individual cancer burden (AYLL) and lowest research spending, lie in the top left quadrant.

In Figure 3 AYLL is shown plotted against percentage of NCRI research spending. In this diagram, tumour sites cluster rather differently from Figure 2. The dashed lines show the means of percentage of NCRI spending and AYLL, for the 17 tumour sites shown. The top left quadrant contains those cancers with the highest AYLL and the lowest research spending, which might therefore be considered ‘Cinderella’ cancer sites. This diagram has several notable features. The positions of breast cancer and leukaemia, both of which have roughly the average AYLL, indicate a considerable excess of research spending over individual cancer burden. A higher research spend relative to AYLL is also directed at colo-rectal and prostate cancers.

Of the four ‘Cinderella’ cancers, cervix is notable because of the high AYLL despite cervical screening. Melanoma is noteworthy because the percentage YLL is relatively modest for this tumour type, accounting for only 1.4% of YLL, whereas the AYLL of just over 15 years indicates that the impact per patient is rather high. It is also increasing in incidence. However, perhaps the most striking of all is that tumours of the brain and CNS have the highest AYLL of all 17 tumour sites, but a rather modest 1.5% of NCRI research spending. The ratio of research spending to AYLL is shown in Table 4.

## DISCUSSION

Using the YLL parameter, to represent population burden from cancer, emphasises the discrepancy between research spending and mortality. Although slightly more difficult to compute than simple percentage mortality, this parameter has added value in demonstrating the effect on the population for individual tumour sites. The absolute differences between YLL and mortality are comparatively small except for colo-rectal and breast cancers, and to a lesser extent bladder and lung cancers. However, the relative differences are much larger for many of the less common tumours (Table 1). Consideration of YLL as a survival parameter might therefore be important if cancer deaths from individual tumour sites are to be used to make decisions concerning research support. The biggest difference between the two indicators is seen for tumours of the brain and CNS, and it is typically difficult to obtain research funding in this area.

It may be that other public health decisions, such as the provision of hospice care, would benefit from consideration of YLL as a mortality indicator. For example, the biggest difference between YLL and simple mortality applies to CNS and cervix cancers, which have a substantial incidence in younger patients. This might suggest the need to consider palliative care resources for younger patients. These two tumour types also account for the highest individual cancer burden, quantified by AYLL. Other measures of social and personal burden, including quality of life, might also be considered as part of a comprehensive review of services.

The different indicators of cancer deaths, and cancer burden, show different aspects of mortality, and are complementary. Analysis of these together can identify tumour types with extreme impact, either on society, for example through a high incidence, or on individual patients as a result of relatively low cure rates. Extreme tumour types, expressed by either statistic, may need special consideration.

This analysis illustrates the value of considering different mortality statistics, which include measures of the burden of cancer deaths on both the population and individual patients. It also demonstrates that inequity in resourcing research goes well beyond the underprovision for lung cancer research. Overall, this analysis suggests that a more subtle and comprehensive calculation of mortality statistics would be useful in relation to research funding, and debate on public health issues.

## Figures and Tables

**Table 1:** Percentage mortality and percentage of YLL for 17 specific tumour sites

**Site**	**% YLL**	**% Mortality**	**% YLL/% mortality**
Brain+CNS	4.1	2.3	1.78
Cervix	2.0	1.3	1.53
Ovary	4.3	3.0	1.44
Melanoma	1.7	1.3	1.33
Leukaemia	3.6	3.0	1.20
Breast	15.5	13.0	1.19
NHL	4.2	3.6	1.18
Kidney	2.4	2.1	1.13
Pancreas	4.3	4.1	1.05
Lung	21.7	20.9	1.04
Oesophagus	3.2	3.2	0.99
Stomach	6.2	6.4	0.98
Uterus	1.7	1.7	0.97
Myeloma	1.9	2.1	0.91
Colon+rectum (+anus)	14.5	16.5	0.87
Bladder	3.4	5.3	0.65
Prostate	5.5	10.2	0.54
			
Total	100	100	

The relative difference between the two parameters of mortality is given by the ratio % YLL/% mortality. Sites are shown in order of this ratio. Figures are presented only for the 17 specified sites, and are therefore normalized to add up to 100%. Data for head and neck cancer, liver cancer and ‘other’ unspecified tumours are not shown, because YLL data are not available for these sites (data from the East Anglian Cancer Registry). YLL=years of life lost; AYLL=avereage years of life lost.

**Table 2:** Percentage of YLL and percentage of NCRI spending, for 17 tumour sites

**Site**	**% YLL**	**% NCRI spend**	**% YLL/% NCRI spend**
Lung	18.2	3.5	5.2
Pancreas	3.6	1.0	3.6
**Stomach**	**5.2**	**1.5**	**3.5**
**Uterus**	**1.4**	**0.5**	**2.8**
Oesophagus	2.7	1.0	2.7
**Brain+CNS**	**3.4**	**1.5**	**2.3**
Bladder	2.9	1.5	1.9
Kidney	2.0	1.5	1.3
Myeloma	1.6	1.5	1.1
Colon+rectum (+anus)	12.0	12.0	1.0
Breast	13.0	18.0	0.7
NHL	3.5	5.5	0.6
Ovary	3.6	6.0	0.6
Prostate	4.7	8.5	0.5
Cervix	1.7	3.5	0.5
Melanoma	1.4	3.0	0.5
Leukaemia	3.0	17.5	0.2
			
Total	83.9	87.5	

The ratio of the two gives an indication of the difference between population burden of deaths from the individual cancers and the research spending on that tumour group. Sites are shown in order of this ratio. For comparison, percentage mortality divided by percentage of NCRI spending is also shown. For the top two sites, lung and pancreas, the ratio of % YLL to research spending is less than the equivalent figure using % mortality. However, for cancers of the stomach, uterus and CNS (shown in bold), the opposite is true, and % YLL exceeds the % of spending on those cancer sites. Data for head and neck cancer, liver cancer and ‘other’ unspecified tumours are not shown, because YLL data are not available for these sites. YLL=years of life lost; AYLL=avereage years of life lost; NCRI=National Cancer Research Institute.

**Table 3:** The AYLL per patient, for 17 tumour sites, in order of descending AYLL

**Site**	**AYLL (years)**	**% YLL**
Brain+CNS	20.1	3.4
Cervix	17.3	1.7
Ovary	16.3	3.6
Melanoma	15.1	1.4
Leukaemia	13.6	3.0
Breast	13.5	13.0
NHL	13.3	3.2
Kidney	12.8	2.0
Pancreas	12.0	3.6
Lung	11.8	18.2
Oesophagus	11.2	2.7
Stomach	11.1	5.2
Uterus	11.0	1.4
Myeloma	10.3	1.6
Colon+rectum (+anus)	9.8	12.0
Bladder	7.3	2.9
Prostate	6.1	4.7
		
Total	83.9%	

The variation in cancer burden per affected patient varies dramatically according to tumour type. The mean AYLL is 12.5 years. The % YLL for each site is shown for comparison (as Table 2). YLL=years of life lost; AYLL=avereage years of life lost.

**Table 4:** Relationship of research spending to the burden of death to individual cancer patients

**Site**	**Annual NCRI research spending (£10^6^)**	**AYLL (years)**	**Annual NCRI research spending/AYLL (£10^6^/year)**
Uterus	1.25	11.0	0.1
Brain+CNS	3.75	20.1	0.2
Pancreas	2.5	12.0	0.2
Oesophagus	2.5	11.2	0.2
Kidney	3.75	12.8	0.3
Stomach	3.75	11.1	0.3
Myeloma	3.75	10.3	0.4
Cervix	8.75	17.3	0.5
Melanoma	7.5	15.1	0.5
Bladder	3.75	7.3	0.5
Lung	8.75	11.8	0.7
Ovary	15.0	16.3	0.9
NHL	13.75	13.3	1.0
Colon+rectum (+anus)	30.0	9.8	3.1
Leukaemia	43.75	13.6	3.2
Breast	45.0	13.5	3.3
Prostate	21.25	6.1	3.5
			
Liver	3.75		
Head and neck	5.0		
Other	25.0		
			
Total	∼£250		

Total NCRI spending amounts to approximately £250 million per year (NCRI, 2002). The factor Annual NCRI research spending divided by AYLL represents the research spending per YLL per patient with each cancer type. Figures for tumours of liver, head and neck and ‘other’, which appear in the NCRI report, are shown. Sites are shown in ascending order of spending per YLL. YLL=years of life lost; AYLL=avereage years of life lost; NCRI=National Cancer Research Institute.
